# Impacts of Ambient Temperatures on Pediatric Anemia in Sub-Saharan Africa: A Regional Ecological Study

**DOI:** 10.3390/ijerph22091364

**Published:** 2025-08-30

**Authors:** Muhammad A. Saeed, Adeena Zaidi, Mohammad R. Saeed, Harris Khokhar, Binish Arif Sultan, Sami Khan, Adam Dawer, Haris Majeed

**Affiliations:** 1 Department of Advanced Academics, Johns Hopkins University, Washington, DC 20001, USA; 2 PRX Research, Dallas Regional Medical Center, Mesquite, TX 75149, USA; 3 Department of Neuroscience, School or Brain and Behavioral Sciences, The University of Texas at Dallas, Richardson, TX 75080, USA; 4 Department of Pathology, Sindh Medical College, Jinnah Sindh Medical University, Karachi 75510, Pakistan; 5 University Health Network, University of Toronto, Toronto, ON M5G 2C4, Canada

**Keywords:** anemia, pediatric/childhood, temperatures, Africa, low-income, linear regression model

## Abstract

Anemia has been a growing concern for the pediatric population in sub-Saharan Africa. Emerging risk factors for anemia under five years of age in low-income countries are multifaceted, including infectious diseases, nutritional deficiencies, hidden hunger, and various economic determinants, and its health burdens include childhood stunting and reduced cognitive function diminished school performance in children. However, the influence of climatic factors, particularly ambient temperatures, on pediatric anemia remains understudied. In this population-based study, we assess the region-specific associations between pediatric anemia and ambient temperatures in 43 countries in Africa from 2000 to 2019. Using generalized linear regression models (upon adjusting for covariates), we found that the risk of temperatures on pediatric anemia varies across four African regions, whereby the Central and Southern African regions have a positive association between pediatric anemia and ambient temperatures, and Western and Eastern regions are negatively affected. The study aims to provide evidence to stakeholders to curtail the onset of pediatric anemia in high-risk African regions to set up key interventions based on the sustainability goals set by the World Health Organization.

## 1. Introduction

Anemia is characterized by a hemoglobin level below the fifth percentile for a given age [1]. With nearly 1.92 billion people, or one-fourth of the world’s population, affected in 2021, anemia is a widely prevalent blood condition with an increase of 420 million occurrences over the past few decades [2]. Children have the highest prevalence of anemia, and the World Health Organization (WHO) reported that around 40% of children aged 6–59 months had anemia globally [3]. In Africa, during 2019, nearly 103 million children were affected by anemia [3]. Furthermore, anemia reached an economic burden of US$ 14,535 per patient per annum, which is 54% higher than the $9451 average cost for non-anemic patients [4].

Previous studies have described the complex and multifaceted etiology of pediatric anemia to include proximal determinants, such as low socioeconomic status [5] and maternal education [6], as well as immediate causes, such as nutritional deficiencies [7] and air pollution [8]. However, there is a paucity of the literature on temperature change as a risk factor for anemia [9]. Globally, Africa is one of the most susceptible regions to climate change, with ambient temperatures on the rise [10]. It is still unclear whether global warming affects pediatric anemia, particularly in low- and middle-income countries (LMIC) [9]. However, children, being a vulnerable group, have a more specific requirement than adults for metabolism and physiology, thereby having a higher chance of being harmed by environmental hazards [11]. A past study also showed that for each 1 °C increase in annual temperature, the odds of anemia prevalence rise by 1.138 [95% CI: 1.134–1.142] [9]. Moreover, African countries with higher temperatures report a higher prevalence of pediatric anemia, whereas those with lower temperatures show lower anemia rates [9]. This association underscores the importance of focusing on temperature as a critical risk factor for pediatric anemia, and the association between temperature and anemia prevalence remained significant, even after controlling for the confounding factors such as household wealth and malaria endemicity [9].

While the true economic burden across Africa is difficult to measure, the projected increase in pediatric anemia is 7597 per 100,000 people annually, showing a drastic increase from baseline [9]. The findings serve as a basis for creating robust policies to mitigate the increasing burden of pediatric anemia. However, due to the inadequate reporting of disease and its surveillance, there may be a knowledge gap [12], and these findings emphasize the need to incorporate climate-related variables into child health policies and anemia prevention efforts across Africa [9]. This study aims to determine the regional differences between temperatures and pediatric anemia rates in Africa. Therefore, it is important to understand the potential health concerns of the long-term impacts of environmental changes, specifically rising temperatures in children.

## 2. Materials and Methods

This is an ecological study using repeated cross-sectional data for Africa from 2000 to 2019. This study included the pediatric population (children < 5 years of age) data for anemia from 43 African nations. The countries were segregated into four regions and divided by geographical location within sub-Saharan Africa (Table 1) due to their distinct geographical, climatic, and socio-political characteristics, allowing for meaningful comparison of regional trends in pediatric anemia and temperature. Northern Africa was excluded due to insufficient longitudinal data on pediatric anemia from WHO sources for the 2000–2019 period. The Eastern African region consisted of the following countries: Burundi, Comoros, Eritrea, Ethiopia, Kenya, Madagascar, Malawi, Mauritius, Mozambique, Rwanda, South Sudan, Uganda, United Republic of Tanzania, Zambia, and Zimbabwe. The central region consisted of Angola, Cameroon, Central African Republic, Chad, Congo, Democratic Republic of the Congo, Gabon, and São Tomé and Príncipe. The southern region consisted of Botswana, Eswatini, Lesotho, Namibia, and South Africa. The western region consisted of Benin, Burkina Faso, Cote d’Ivoire, Gambia, Ghana, Guinea, Guinea-Bissau, Liberia, Mali, Mauritania, Niger, Nigeria, Senegal, Sierra Leone, and Togo.

The outcome data was obtained from the World Health Organization (WHO), which compiles nationally reported health statistics submitted by each country across years to allow for accurate comparisons. Prevalence of pediatric anemia is considered the ratio of the annual number of children with anemia to the country-specific population for children less than 5 years of age. The study allows for the identification of long-term patterns and regional disparities. Because the data was collected at the national level, results are intended to reflect broader regional trends. Aside from our primary outcome, exposure data for country-specific annual mean air temperature data from 2000 to 2019 were obtained from the World Bank [13]. We included covariate data on country-specific annual mean air pollution (specifically NO_2_ in μg/m^3^), cereal yield (in tonnes per hectare), gross domestic product (GDP), prevalence of child stunting, prevalence of reproductive women with anemia (moderate levels), average children’s hemoglobin, and annual trend. These covariates were chosen based on previous research identifying them as key environmental and health-related factors that may affect anemia.

All variables were formatted consistently by country and year to enable comparative analyses across time and region. However, temperature was the main variable in a growing body of climate-health research that identifies heat as a systemic factor affecting metabolic demand, infection rates, and nutritional absorption—all of which are critical for pediatric well-being and development. Additionally, since anemia is influenced by a variety of environmental and nutritional factors, identifying temperature as a primary exposure allows for a clearer interpretation of region-specific trends. Narrowing our analysis to the effect of temperature on pediatric anemia allowed us to isolate a specific environmental factor, temperature, increasing the strength of this study and making it region-specific.

The data used in the study from the WHO is from recognized sources to ensure reliability and comparability and ensure consistency in data quality. We modeled the region-specific associations between the annual number of children and average air temperatures using a generalized linear model: specifically, negative binomial regression using autocorrelation order 1 (i.e., AR1). This is because the outcome’s variance was much greater than its mean, leading to overdispersion of data. Model coefficients were exponentiated to be interpreted as rate ratios (RR) for each 1 °C rise in annual mean air temperatures. Confidence intervals of 95% were evaluated at *p* < 0.05 using Student’s two-sided *t*-tests. Microsoft Excel (v.2021) and RStudio (v.4.1.1) were used for computation, analyses, and figure composition. The regression model was chosen to handle time-based data where values may be related across consecutive years, helping to reduce potential bias in long-term trend analysis. Missing data points for certain countries and years were excluded from the analysis to maintain consistency in the model. While this study focused on temperature, additional confounders were fixed, and future research may consider expanded multivariable approaches.

Additionally, generative artificial intelligence (GenAI) or any other AI tools have not been used for any part of this paper.

## 3. Results

The study data from 2000 to 2019, for 43 countries, within four regions of Africa, shows that there is a total of 1,775,405,490 pediatric anemia cases as reported by the WHO. Out of the four African regions, as defined earlier, Western Africa bore the highest proportion of pediatric anemia cases (44.3%), followed by Eastern Africa with 34.6%, Central Africa with 18.2%, and lastly, Southern Africa had the lowest proportion (2.9%).

**Figure 1:**
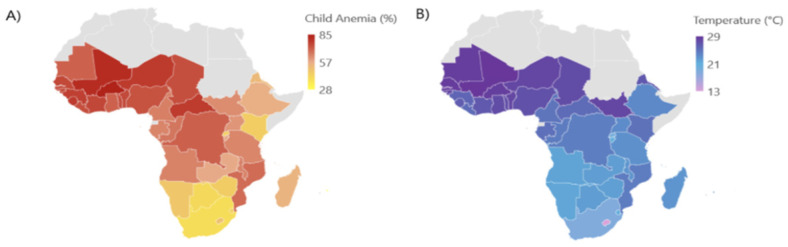
(**A**) Annual country-specific child (<5 years) anemia prevalence; (**B**) annual mean temperatures from 2000 to 2019.

Geographically, the prevalence of pediatric anemia is observed to be the highest in Western and Eastern Africa (Figure 1A). Similarly, annual mean air temperatures were highest in Western Africa, reaching 27.7 °C, followed by Central Africa at 25.0 °C, Eastern Africa with 23.3 °C, and Southern Africa with the lowest mean air temperature at 18.0 °C throughout the 2000 to 2019 period (Figure 1B and Table 2).

For Central Africa, an RR of 1.023 indicates a 2.3% increase in anemia prevalence for every 1 °C rise in temperature, upon controlling for other covariates. Similarly, for Southern Africa, an RR of 1.005 suggests a 0.5% increase per 1 °C rise. Conversely, the Western RR of 0.989 and Eastern Africa RR of 0.993 show a 1.1% and 0.7% decrease, respectively, in anemia prevalence per 1 °C rise (Table 3). It confirms the variability in the relationship of temperature being a statistically significant predictor of pediatric anemia across all four African regions. Overall, Central Africa had the strongest effect of annual mean air temperatures on pediatric anemia from 2000 to 2019.

## 4. Discussion

Our findings from the WHO between 2000 and 2019 show that Eastern and Western Africa demonstrated an inverse relationship between the prevalence of pediatric anemia and temperature, whereas Central and Southern Africa exhibited a direct relationship. One previous study also found regional differences in Africa with an association between ambient heat and child mortality [14]. This past study also showed a 1.27 risk ratio [95% CI: 1.19–1.36] for child mortality in Eastern Africa associated with heat exposure while also showing a 0.92 risk ratio [95% CI: 0.88–0.97] for child mortality in Southern Ghana, Côte d’Ivoire, and Nigeria—all countries located in Western Africa [14]. The study by Brimicombe et al. is consistent with ours, in that there are regional differences in the association between temperature and child health [14].

The biological pathways linking temperature to pediatric anemia are complex, involving various environmental effects [9]. While our study focuses on environmental determinants of pediatric anemia, particularly ambient temperature, we acknowledge that hemoglobinopathies, especially sickle cell disease, are a contributor to anemia in African children. The interaction between temperature and hemoglobin disorders is not well established in the current literature. However, it is plausible that extreme temperatures may exacerbate clinical symptoms in children with sickle cell disease, such as vaso-occlusive crises triggered by dehydration or thermal stress. Further research is needed to explore these potential interactions. One plausible mechanism is that warmer temperatures increase parasite spread, heightening malaria risk and consequently raising anemia rates, with studies suggesting that this effect on pediatric anemia is significantly mediated through malaria infection [15,16,17]. Moreover, the prevention of anemia was shown to result from avoidance of exposure to heat, which further links increased heat with a rise in anemia [18]. Although previous studies have linked the lack of fluid retention with the severity of anemia, further research is needed to reach a definitive conclusion [19]. A history of diarrhea has also been identified as a risk factor for pediatric anemia, with an adjusted odds ratio of 2.44 [95% CI: 1.03–3.85], indicating a significant association between diarrhea and anemia [20]. Additionally, high temperatures in combination with drought can worsen water shortages, causing poor sanitation and hygiene, contributing to environmental enteric dysfunction—a significant risk factor for pediatric anemia [21,22].

Interestingly, there is a limitation in the availability of data from Africa on the amount of time children spend outdoors and their exposure to outside temperatures, which can contribute to the applicability of the relationship. A study conducted by Iqbal et al. highlighted the behavioral differences in the school-aged pediatric populations in rural areas who spend more time outdoors, and the increased outdoor exposure, particularly in warmer climates, may heighten vulnerability [23]. Therefore, the differing prevalence rates of pediatric anemia across African regions may partly reflect variations in the amount of time pediatric populations are likely to spend outdoors, and differences in outdoor school activity and access to shaded environments may influence pediatric susceptibility to heat-related health effects. While these findings do not directly align with our dataset, they offer additional insight into the idea that regional differences in pediatric health outcomes, such as anemia, which in the future may be shaped by temperature exposure and related behaviors.

Another factor that can affect the anemia prevalence independently of the temperature variability is the availability, timing, and quality of public health interventions. Furthermore, nutritional aid programs, when unevenly distributed across regions, may impact anemia trends independently of environmental exposure, such as temperature. Understanding these interacting elements is essential for disentangling the role of temperature from broader health systems and policy factors.

Additionally, systematic factors, such as the health consequences of cold temperatures, should also be considered alongside biological responses to develop a better understanding of anemia prevalence, its health consequences, and the burden of disease. A previous study found that cold temperatures affect child health by increasing severe neonatal infections, such as pneumonia and sepsis, which can further exacerbate an infant’s physiological susceptibility to heat and cold in Africa [24]. The impacts of climate change on temperature-related neonatal mortality were largest in countries that had relatively high baseline neonatal mortality rates and experienced large temperature increases due to climate change (Sierra Leone, Ethiopia, Liberia, Mali, Guinea, Benin, Cameroon, Nigeria, Angola, Timor-Leste, Haiti) [24]. Additionally, a past study indicates that child mortality in South Africa was associated with both cold and heat, and the total attributable mortality was 3.4%, mostly from cold (3.0%) rather than heat (0.4%), using confidence intervals determined by an approximate parametric bootstrap estimator [25,26].

The temperature data of Central Africa suggested that it is particularly vulnerable to temperature-driven increases in pediatric anemia prevalence. This effect may indicate that the health impact of rising temperatures is more pronounced in certain regions due to underlying vulnerabilities. Conversely, the inverse trends observed in Western and Eastern Africa may be shaped by localized interventions or unmeasured regional factors that buffer the effects of heat exposure. Additionally, infections are a risk factor for pediatric anemia [8], and cold temperatures have been shown to increase pneumonia risk by 1.06 times [95% CI: 0.98–1.14] in children [27], suggesting that colder temperatures are also associated with increased infection rates. In summary, understanding how temperatures are directly linked to pediatric anemia in low-income regions such as Sub-Saharan Africa is essential for addressing associated health issues [28]. Specifically, anemia rates in Western Africa, Central Africa, and parts of Eastern Africa have been affected by other factors, which may have mediated the associations between annual mean temperature and pediatric anemia by 11.40% and 9.74%, respectively [9].

## 5. Limitations

Currently, the pediatric datasets available are varied. Longitudinal data and census data, such as the one used in this study, do not provide details as to whether children surveyed are repeated. Because the data consist of annual, country-level compilations rather than individual-level panel data, it was not possible to determine if the same children were repeatedly surveyed across years. Ecological fallacy exists, and it must be noted that associations observed at the country level may not reflect individual-level relationships. Therefore, a panel data analysis was not feasible, and our models are based on annual prevalence rates. Furthermore, varying degrees of patient accounting across rural and urban hospitals in Africa limit the depth to which a retrospective analysis can be made. Nonetheless, the data we do have access to and the analysis we conducted provide clear proof of the association between ambient temperature and pediatric anemia.

This study was also limited by the data being generalized to regions of Africa, which did not allow for the consideration of specific disparities such as healthcare access [29], access to adequate nutrition [30,31], and sufficient sanitation [32], in turn leading to increased rates of pediatric anemia that may have confounded the results. Additionally, since topographic elevation is associated with temperature [33], the generalization of data to large regions may have impaired the temperature data collected, due to the possibility of topographic variations within each region affecting the occurrence of ambient temperatures. The study did not consider other regional risk factors, such as maternal education [34] and access to water within households [32], which may also contribute to an increase in pediatric anemia. Northern Africa was also not assessed in this study, since there is limited data regarding pediatric anemia prevalence over time in this region. Additionally, though it was discussed that the regional differences in the effects of ambient temperature on anemia prevalence may partly be attributed to the differing amounts of time children may spend outdoors in each region, data to support this is still limited. Further studies are needed to determine confounding variables, such as the impact of region-specific activities, conflict, and cultural nuances, which may also compound the impacts on increasing anemia in children under five years old. The lack of consistent health surveillance infrastructure across all regions also limits the generalizability of some regional comparisons [35].

Furthermore, disparities in pediatric healthcare infrastructure and uneven regional capacity to respond to climate change likely contribute to differences in how temperature affects anemia risk in children under five [36]. In regions with well-established public health systems, children may have better access to iron supplements, malaria prevention tools, and nutrition programs that reduce temperature-related health risks [36]. In contrast, children in regions with limited healthcare access may be more vulnerable to temperature-related infections and anemia, due to inadequate preventive care [36]. This imbalance may partially explain why the strength of the temperature–anemia association differs across African regions. While this limitation introduces some variability, it does not undermine the integrity of our results, which are based on consistent national-level data and demonstrate clear region-specific patterns. Nonetheless, the data collected in the study can support a regional association between ambient temperatures and the prevalence of pediatric anemia in Africa.

## 6. Conclusions

The urgent public health crisis in Africa demands evidence-based recommendations specific to each region in Africa. Anemia already poses significant threats to the health of children under five years old, and with the unknown long-term impacts of global warming on high-risk regions in Africa, our findings aim to bolster the ability of regional organizations and policymakers to curb the crisis at hand. Temperature trends do not affect all regions uniformly, with some areas showing a positive association, while others show an inverse pattern. Central Africa experiences a particularly strong link between higher temperatures and increased pediatric anemia. Conversely, Western and Eastern Africa show a slightly negative association, suggesting that temperature may interact differently with local factors in those regions. Behavioral patterns, such as increased outdoor exposure among children in warmer climates, may further mediate anemia risk. Implementing region-specific policies and programs that address the distinct risk factors in each area is crucial for effectively reducing the burden of pediatric anemia across the continent. This study brings new evidence of regional differences in how temperature affects the prevalence of anemia differently in different regions of Africa. In addition to regional trends, other environmental stressors like drought and poor sanitation should be explored. To support intervention efforts, it is important to prioritize investments in pediatric health infrastructure, particularly in rural and underserved regions where children are most vulnerable to environmental health threats. Strengthening partnerships between local health ministries, climate researchers, and international organizations can also increase the development of solutions that reflect both environmental and sociodemographic realities. Future research should also investigate whether temperature-related effects differ by sex, education level, or other demographic factors to support more targeted interventions. Incorporating climate variables into anemia risk prediction models could help allocate prevention resources more effectively in high-risk zones. Additional studies can also employ projection models to show how the findings of this study may change in the future with global temperature changes.

## Figures and Tables

**Table 1:** Shows the number of children per annum with anemia for each country and their corresponding region from 2000 to 2019.

Region	Country	Number of Children with Anemia
Eastern (*n* = 613,660,310)	Burundi	17,626,800
	Comoros	1,187,090
	Eritrea	4,714,000
	Ethiopia	158,345,000
	Kenya	57,797,000
	Madagascar	36,580,000
	Malawi	32,383,000
	Mauritius	459,320
	Mozambique	56,049,000
	Rwanda	13,688,100
	South Sudan	18,374,200
	Uganda	74,517,000
	United Republic of Tanzania	96,414,000
	Zambia	27,740,000
	Zimbabwe	17,785,800
Central (*n* = 323,793,130)	Angola	55,729,000
	Cameroon	43,674,000
	Central African Republic	10,984,700
	Chad	33,943,000
	Congo	8,928,000
	Democratic Republic of the Congo	167,170,000
	Gabon	3,003,400
	Sao Tome and Principe	361,030
Southern (*n* = 50,795,450)	Botswana	2,020,990
	Eswatini	1,298,760
	Lesotho	2,545,000
	Namibia	2,724,700
	South Africa	42,206,000
Western (*n* = 787,156,600)	Benin	22,949,200
	Burkina Faso	47,534,000
	Cote d’Ivoire	50,802,000
	Gambia	4,291,700
	Ghana	49,904,000
	Guinea	27,065,000
	Guinea-Bissau	3,718,300
	Liberia	9,127,400
	Mali	46,473,000
	Mauritania	7,693,400
	Niger	53,040,000
	Nigeria	401,095,000
	Senegal	32,168,000
	Sierra Leone	15,842,100
	Togo	15,453,500
Total		1,775,405,490

**Table 2:** Regional-specific mean air temperatures from 2000 to 2019.

Region	Mean Air Temperature (2000–2019)
Western	27.7 °C
Eastern	23.3 °C
Central	25.0 °C
Southern	18.0 °C

**Table 3:** Shows that for each 1 °C rise in air temperature, the RR for pediatric anemia in Western [0.989 (*p* = 0.0013)], Eastern [0.993 (*p* = 0.001)], Central [1.023 (*p* < 0.0001)], and Southern Africa [1.005 (*p* = 0.006)] had differing magnitude associations.

African Region	Number of Cases	RR (95% CI)
Western	787,156,600	0.989 (*p* = 0.0013)
Eastern	613,660,310	0.993 (*p* = 0.001)
Central	323,793,130	1.023 (*p* < 0.0001)
Southern	50,795,450	1.005 (*p* = 0.006)

## Data Availability

The original contributions presented in this study are included in the article. Further inquiries can be directed to the corresponding author.

## Abbreviations

The following abbreviations are used in this manuscript:

WHO	World Health Organization
US	United States
LMIC	Low- and middle-income countries
CI	Confidence interval
GDP	Gross domestic product
RR	Rate ratio

## References

[1] Janus J., Moerschel S.K. (2010). Evaluation of anemia in children. Am. Fam. Physician.

[2] Institute for Health Metrics and Evaluation (2023). The Lancet: New Study Reveals Global Anemia Cases Remain Persistently High Among Women and Children. Anemia Rates Decline for Men. [Internet]. https://www.healthdata.org/news-events/newsroom/news-releases/lancet-new-study-reveals-global-anemia-cases-remain-persistently#:~:text=Topics&text=One%2Dfourth%20of%20the%20global.

[3] World Health Organization (2023). Anaemia [Internet]. https://www.who.int/news-room/fact-sheets/detail/anaemia.

[4] Nissenson A.R., Wade S., Goodnough T., Knight K., Dubois R.W. (2005). Economic Burden of Anemia in an Insured Population. J. Manag. Care Pharm..

[5] Yang F., Liu X., Zha P. (2018). Trends in Socioeconomic Inequalities and Prevalence of Anemia Among Children and Nonpregnant Women in Low- and Middle-Income Countries. JAMA Netw. Open.

[6] Khan J.R., Awan N., Misu F. (2016). Determinants of anemia among 6–59 months aged children in Bangladesh: Evidence from nationally representative data. BMC Pediatr..

[7] Martinez-Torres V., Torres N., Davis J.A., Corrales-Medina F.F. (2023). Anemia and Associated Risk Factors in Pediatric Patients. Pediatric Health Med. Ther..

[8] Amegbor P.M. (2022). Early-life environmental exposures and anaemia among children under age five in Sub-Saharan Africa: An insight from the Demographic & Health Surveys. Sci. Total Environ..

[9] Zhu Y., He C., Gasparrini A., Vicedo-Cabrera A.M., Liu C., Bachwenkizi J., Zhou L., Cheng Y., Kan L., Chen R. (2023). Global warming may significantly increase childhood anemia burden in sub-Saharan Africa. One Earth.

[10] Moyo E., Nhari L.G., Moyo P., Murewanhema G., Dzinamarira T. (2023). Health effects of climate change in Africa: A call for an improved implementation of prevention measures. Eco-Environ. Health.

[11] UNICEF (2025). Children’s Unique Vulnerabilities to Environmental Hazards [Internet].

[12] Mremi I.R., George J., Rumisha S.F., Sindato C., Kimera S.I., Mboera L.E.G. (2021). Twenty years of integrated disease surveillance and response in Sub-Saharan Africa: Challenges and opportunities for effective management of infectious disease epidemics. One Health Outlook.

[13] Palinatx (2022). Mean Temperature for Countries by Year 1901–2022 [Dataset]. Kaggle. https://www.kaggle.com/datasets/palinatx/mean-temperature-for-countries-by-year-2014-2022.

[14] Brimicombe C., Wieser K., Monthaler T., Jackson D., De Bont J., Chersich M.F., Otto I.M. (2024). Effects of ambient heat exposure on risk of all-cause mortality in children younger than 5 years in Africa: A pooled time-series analysis. Lancet Planet. Health.

[15] Short E.E., Caminade C., Thomas B.N. (2017). Climate Change Contribution to the Emergence or Re-Emergence of Parasitic Diseases. Infect. Dis..

[16] Kuile F.O., Terlouw D.J., Kariuki S.K., Phillips-Howard P.A., Mirel L.B., Hawley W.A., Friedman J.F., Shi Y.P., Kolczak M.S., Lal A.A. (2003). Impact of permethrin-treated bed nets on malaria, anemia, and growth in infants in an area of intense perennial malaria transmission in western Kenya. Am. J. Trop. Med. Hyg..

[17] Iannotti L.L., Tielsch J.M., Black M.M., Black R.E. (2006). Iron supplementation in early childhood: Health benefits and risks. Am. J. Clin. Nutr..

[18] Awuah R.B., Colecraft E.K., Wilson M.L., Adjorlolo L.K., Lambrecht N.J., Nyantakyi-Frimpong H., Jones A.D. (2021). Perceptions and beliefs about anaemia: A qualitative study in three agroecological regions of Ghana. Matern. Child. Nutr..

[19] Hung S.C., Kuo K.L., Peng C.H., Wu C.H., Wang Y.C., Tarng D.C. (2015). Association of fluid retention with anemia and clinical outcomes among patients with chronic kidney disease. J. Am. Heart Assoc..

[20] Azmeraw M., Kassaw A., Habtegiorgis S.D., Tigabu A., Amare A.T., Mekuria K., Temesgen D., Zemariam A.B., Kerebeh G., Bantie B. (2023). Prevalence of anemia and its associated factors among children aged 6-23 months, in Ethiopia: A systematic review and meta analysis. BMC Public Health.

[21] Humphrey J.H., Jones A.D., Manges A., Mangwadu G., Maluccio J.A., Mbuya M.N.N., Moulton L.H., Ntozini R., Prendergast A.J., The Sanitation Hygiene Infant Nutrition Efficacy (SHINE) Trial Team (2015). The Sanitation Hygiene Infant Nutrition Efficacy (SHINE) Trial: Rationale, Design, and Methods. Clin. Infect. Dis..

[22] Wilson S.E., Rogers L.M., Garcia-Casal M.N., Barreix M., Bosman A., Cunningham J., Goga A., Montresor A., Tuncalp O. (2023). Comprehensive framework for integrated action on the prevention, diagnosis, and management of anemia: An introduction. Ann. N. Y. Acad. Sci..

[23] Li J., Haragakiza J.D., Fisher D., Pronyuk K., Zhao L. (2024). Current status of malaria control and elimination in Africa: Epidemiology, diagnosis, treatment, progress and challenges. J. Epidemiol. Glob. Health.

[24] Dimitrova A., Dimitrova A., Mengel M., Gasparrini A., Lotze-Campen H., Gabrysch S. (2024). Temperature-related neonatal deaths attributable to climate change in 29 low- and middle-income countries. Nat. Commun..

[25] Scovronick N., Sera F., Acquaotta F., Garzena D., Fratianni S., Wright C.Y., Gasparrini A. (2018). The association between ambient temperature and mortality in South Africa: A time-series analysis. Environ. Res..

[26] Tobías A., Armstrong B., Gasparrini A. (2017). Brief Report: Investigating Uncertainty in the Minimum Mortality Temperature. Epidemiology.

[27] Makrufardi F., Triasih R., Nurnaningsih N., Chung K.F., Lin S.-C., Chuang H.-C. (2024). Extreme temperatures increase the risk of pediatric pneumonia: A systematic review and meta-analysis. Front. Pediatr..

[28] Brehm R., South A., George E.C. (2023). Use of point-of-care haemoglobin tests to diagnose childhood anaemia in low- and middle-income countries: A systematic review. Trop. Med. Int. Health.

[29] Kang N., Wang R., Lu H., Onyai F., Tang M., Tong M., Ni X., Zhong M., Deng J., Dong Y. (2024). Burden of Child Anemia Attributable to Fine Particulate Matters Brought by Sand Dusts in Low- and Middle-Income Countries. Environ. Sci. Technol..

[30] Shimanda P.P., Amukugo H.J., Norström F. (2020). Socioeconomic factors associated with anemia among children aged 6-59 months in Namibia. J. Public Health Afr..

[31] Nkulikiyinka R., Binagwaho A., Palmer K. (2015). The changing importance of key factors associated with anaemia in 6- to 59-month-old children in a sub-Saharan African setting where malaria is on the decline: Analysis of the Rwanda Demographic and Health Survey 2010. Trop. Med. Int. Health.

[32] Kothari M.T., Coile A., Huestis A., Pullum T., Garrett D., Engmann C. (2019). Exploring associations between water, sanitation, and anemia through 47 nationally representative demographic and health surveys. Ann. N. Y. Acad. Sci..

[33] Ogwang B.A., Chen H., Li X., Gao C. (2014). The Influence of Topography on East African October to December Climate: Sensitivity Experiments with RegCM4. Adv. Meteorol..

[34] Choi H.J., Lee H.J., Jang H.B., Park J.Y., Kang J.H., Park K.H., Song J. (2011). Effects of maternal education on diet, anemia, and iron deficiency in Korean school-aged children. BMC Public Health.

[35] Bacon E., Budney G., Bondy J., Kahn M.G., McCormick E.V., Steiner J.F., Tabano D., Waxmonsky J.A., Zucker R., Davidson A.J. (2019). Developing a Regional Distributed Data Network for Surveillance of Chronic Health Conditions: The Colorado Health Observation Regional Data Service. J. Public Health Manag. Pract..

[36] Neuberger A., Okebe J., Yahav D., Paul M. (2016). Oral iron supplements for children in malaria-endemic areas. Cochrane Database Syst. Rev..

